# How a Good Sleep Predicts Life Satisfaction: The Role of Zero-Sum Beliefs About Happiness

**DOI:** 10.3389/fpsyg.2018.01589

**Published:** 2018-08-28

**Authors:** Ji-eun Shin, Jung Ki Kim

**Affiliations:** ^1^College of Liberal Studies, Seoul National University, Seoul, South Korea; ^2^Division of Humanities and Social Sciences, Pohang University of Science and Technology, Pohang, South Korea

**Keywords:** sleep quality, life satisfaction, happiness, zero-sum, lay belief

## Abstract

Sleep, although a vital aspect of human functioning, has received scant attention in happiness research. This research examines the effect of sleep quality on life satisfaction, and one possible mechanism that bridges the two. One cognitive factor that might tie the relationship between sleep and life satisfaction is a belief about the (in) finite nature of happiness (zero-sum belief about happiness; ZBH), a mindset that occurs more under conditions of scarcity. Given the interconnections among experiences prompted by various types of scarcity (e.g., financial and calorie), we predicted that deprived cognitive resource caused by poor sleep may activate the ZBH, thereby hurting one’s life satisfaction. As expected, we found that sleep quality predicted the participants’ life satisfaction, even controlling for baseline variables. More importantly, this relationship was partially mediated by ZBH. This study opens interesting questions on a relatively unexamined role of non-social predictors, such as sleep, in well-being.

## Introduction

As highly social beings, humans are wired to connect with others. Not surprisingly, social experience is one of the most heavily studied topics in happiness research (Diener and Seligman, 2002; Diener et al., 2018). However, no human beings are constantly with others. For example, the average person spends about one third of her time not interacting with others, sleeping. Although a significant portion of time is spent on sleeping, few have examined *how* this experience relates to happiness (however, see Paunio et al., 2008; Sonnentag et al., 2008; Steptoe et al., 2008). The purpose of this study is to verify the association between sleep and life satisfaction, and investigate one possible cognitive belief that may bridge the two.

Various personal beliefs about happiness affect the actual level of happiness experienced by the person. For instance, those who consider happiness as more relational (Bojanowska and Zalewska, 2016; Shin et al., 2018), controllable (Joshanloo, 2017), and incremental (Tamir et al., 2007) are happier than others. Here we focused on a potentially important, yet unexamined mediating belief – whether happiness exists as a zero-sum state (zero-sum belief about happiness; ZBH). Zero-sum beliefs in general (cf. Von Neumann and Morgenstern, 1944) are based on the assumption that a finite amount of goods exists in the world, in which one’s gain is possible only at the expense of others. Viewed in this way, those with high ZBH are presumed to consider the amount of happiness as fixed (vs. unlimited), amongst people (i.e., if someone gets happier, somebody else might become less happy) as well as across time (i.e., if one is happy now, he or she might become less happy in the future). Such zero-sum beliefs about happiness predict low well-being (Koo and Suh, 2007; Różycka-Tran et al., 2015).

One condition that activates such zero-sum thinking is resource scarcity (Różycka-Tran et al., 2015). The authors noted that resource-deprived mindset may lead individuals to perceive the world as competitive, becoming more prone to zero-sum beliefs. Although scarcity arises in various domains (e.g., financial and calorie), there is a common overlap among the diverse experiences of scarcity (Muraven and Baumeister, 2000; Briers et al., 2006). Sleep replenishes depleted resource (Saper et al., 2005). Conversely, impaired sleep may trigger a sense of scarcity (especially, cognitive), leading people to perceive happiness more in a zero-sum manner. In other words, ZBH would be driven in part by low sleep quality and, consequently lead to a temporary drop in life satisfaction.

In sum, the purpose of this study was to examine whether a good sleep predicts life satisfaction by reducing ZBH. By collecting data at two time points (4-week intervals), we further examined whether the predictive power of sleep on life satisfaction remain significant even after controlling for baseline happiness and other relevant factors (e.g., personality).

## Materials and Methods

### Participants

Two hundred and fifty-seven undergraduate students voluntarily participated and received $5 for their participation. Eighteen participants were excluded due to incomplete responses, leaving a final sample of 239 participants (female = 112; *M*_age_ = 20.61, *SD*_age_ = 1.06). Participants visited the lab twice, and received monetary compensation at the end of the session.

### Measures

The study took place over a 4-week interval. At Time 1, participants signed the consent form and answered the Pittsburgh Sleep Quality Index (PSQI; Buysse et al., 1989), which measures the quality of their sleep during the past month. This questionnaire consists of 19 items categorized into 7 components (e.g., sleep latency and daytime dysfunction). The sum of scores for 7 components (α = 0.79) yielded a single index, ranging from 0 to 21. In our data, the mean PSQI score was seven (*SD* = 1.92), with a range from 3 to 15. To make our data easier to understand, we reversed the coding so that higher numbers indicate better sleep quality.

At Time 1, as control measures, baseline life satisfaction and two conceptually relevant factors (personality, financial status) were assessed. Life satisfaction was measured with a single item (“In general, how satisfied are you with your life?”) on a 7-point scale. This single item life satisfaction measure was found to be reliable and valid (Cheung and Lucas, 2014). Personality was assessed with a 25-item measure of the five-factor (Big 5) model of personality (Brody and Ehrlichman, 1998) on a 7-point scale (1 = *not at all true of me* and 7 = *very true of me*). Based on the correlational findings observed in prior studies (Taylor and McFatter, 2003; Różycka-Tran et al., 2015), we particularly considered extraversion (e.g., talkative, outgoing; α = 0.79), neuroticism (e.g., anxious, worrying; α = 0.85), and agreeableness (e.g., warm, generous; α = 0.71). One’s perceived socioeconomic status (SES), which relates with zero-sum thinking (Różycka-Tran et al., 2015), was measured by a commonly used scale (e.g., Kraus et al., 2009; “What level do you think your household income belongs to?”). Participants placed themselves on a graphical 10-rung ladder, ranging from 1 (*low income*) to 10 (*high income*).

At Time 2, we assessed ZBH as a potential mediator as well as life satisfaction. ZBH was measured with two 7-point scale items (partly adopted from Różycka-Tran et al., 2015). To measure the zero-sum belief amongst people, respondents chose a number that best reflected their belief (1 = if someone becomes happier, it does *not* mean that someone else will become less happy; 7 = if someone becomes happier, its means someone else will become less happy). Zero-sum belief across the time dimension was measured on a scale from 1 (if someone is happy now, it does *not* mean that s/he will be less happy in the future) to 7 (if someone is happy now, it means s/he will be less happy in the future). The two items were averaged (α = 0.81) as a ZBH score (higher score indicating greater zero-sum thinking). When we conducted a pilot study (217 undergraduates voluntarily participated as part of a psychology class) to assess the diagnostic value of ZBH, there was a strong correlation (*r* = 0.68, *p* < 0.001) between ZBH and a previously validated scale of zero-sum axiomatic belief (α = 0.74; Różycka-Tran et al., 2015). Finally, Time 2 Life satisfaction was assessed with the Satisfaction with Life Scale (SWLS; Diener et al., 1985). A sample item reads, “I am satisfied with my life.” Responses were made on a 7-point scale (1 = *strongly disagree*, 7 = *strongly agree*) and the 5 items were averaged (α = 0.87).

## Results

To what extent do people believe that happiness is a zero-sum experience? As shown in **Table 1**, the mean score of ZBH was 3.24 (*SD* = 1.51; 4 being the midpoint), suggesting that happiness was viewed as a fixed, finite experience by a minority of the respondents (21.8% of total participants). As expected, ZBH was related to sleep quality and both Time 1 and Time 2 life satisfaction. Those who suffered from poor sleep endorsed ZBH more strongly, and consistent with prior findings (Paunio et al., 2008), reported lower life satisfaction.

**Table 1:** Descriptive statistics of and correlations between variables.

Variables	*M (SD)*	1	2	3	4	5	6	7	8	9
*Time 1*										
1. Sex (0 = male)	0.47 (0.50)	-								
2. Age	20.61 (1.06)	-0.06	-							
3. Sleep quality	14.00 (1.92)	-0.05	-0.07	-						
4. Life satisfaction (single item)	4.82 (1.24)	-0.04	0.01	0.16^∗^	-					
5. Extraversion	4.25 (1.04)	-0.10	-0.02	0.04	0.29^∗∗∗^	-				
6. Neuroticism	4.04 (1.23)	0.08	0.06	-0.28^∗∗∗^	-0.19^∗∗^	-0.25^∗∗∗^	-			
7. Agreeableness	4.87 (0.81)	-0.08	0.07	0.09	0.19^∗∗^	0.29^∗∗∗^	0.08	-		
8. SES	5.16 (1.86)	-0.06	-0.10	0.09	0.14^∗^	0.04	0.08	0.10	-	
*Time 2*										
9. ZBH	3.24 (1.51)	0.06	0.01	-0.21^∗∗^	-0.14^∗^	-0.18^∗∗^	0.14^∗^	-0.11^†^	-0.14^∗^	-
10. Life satisfaction (SWLS)	4.39 (1.13)	-0.07	-0.06	0.25^∗∗∗^	0.51^∗∗∗^	0.35^∗∗∗^	-0.36^∗∗∗^	0.09	0.27^∗∗∗^	-0.32^∗∗∗^


*^†^*p* < 0.08,^∗^*p* < 0.05, *^∗∗^p* < 0.01, *^∗∗∗^p* < 0.001. SES, socioeconomic status; ZBH, zero-sum beliefs about happiness; SWLS, the satisfaction with life scale.*

Does ZBH play a role in this sleep-happiness relation? A mediation analysis using the PROCESS macro (Hayes, 2013; 5,000 bootstrapped samples) was performed, after mean-centering the variables. With respect to Time 2 life satisfaction, there were significant main effects of sleep quality, *b* (*SE*) = 0.11 (0.04), *p* < 0.01, CI_95_ = [0.04, 0.18], and ZBH, *b* (*SE*) = -0.21 (0.05), *p* < 0.001, CI_95_ = [-0.30, -0.12]. More importantly, the effect of sleep quality on Time 2 life satisfaction was mediated by ZBH, *b* (*SE*) = 0.03 (0.01), *p* < 0.01, CI_95_ = [0.01, 0.06].

**FIGURE 1:**
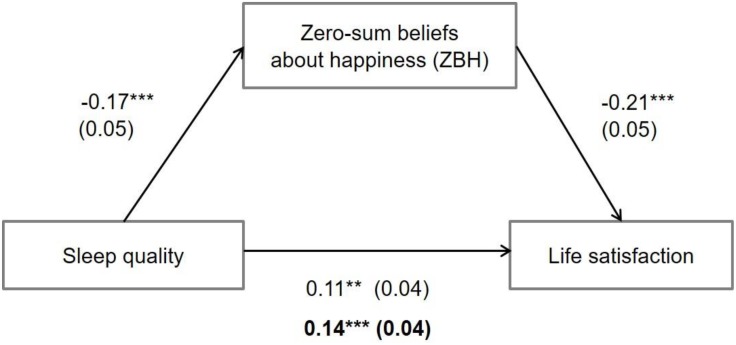
ZBH as a mediator of life satisfaction. Regression coefficients are unstandardized, and the total effect of sleep quality is marked in bold (^∗∗^*p* < 0.01, ^∗∗∗^*p* < 0.001).

As shown in **Figure 1**, those with high sleep quality experienced greater life satisfaction partly because they were less likely to endorse ZBH. These mediation results held after controlling for Time 1 life satisfaction, *b* (*SE*) = 0.03 (0.01), *p* = 0.016, CI_95_ = [0.01, 0.05], and other covariates that were significantly related to main variables (extraversion, neuroticism, and SES), *b* (*SE*) = 0.02 (0.01), *p* = 0.038, CI_95_ = [0.01, 0.05]. Overall, a good sleep seems to predict life satisfaction by reducing a zero-sum mindset.

## Discussion

A significant portion of daily time is spent on sleep. Yet, much remains to be known about how sleep relates to life satisfaction. In this research, we found that those who sleep well are more satisfied with life, controlling for individual characteristics such as personality. Although social relationships are essential for well-being (Diener and Seligman, 2002; Sandstrom and Dunn, 2014), social activity is costly and energy-consuming (Baumeister et al., 2000). This is perhaps why people need a certain amount of time alone, which serves a restorative function (Coplan and Bowker, 2014). Sleep, although far less studied than social experiences, needs more research attention in future happiness research.

More research is needed to clarify how sleep predicts life satisfaction, but we uncovered one possibility. Those who sleep poorly were more likely to view happiness as a zero-sum game. A zero-sum mindset leads people to engage in more social comparison and savor positive experiences less, which eventually lead to less happiness (Koo and Suh, 2007). As many societies become more competitive and market-oriented, sleep is easily regarded as a waste of time (and money). However, sacrificed sleep may create a vicious cycle of making the world appear as a zero-sum competition, which aggravates interpersonal stress. Increasing public awareness of the importance of sleep might have greater societal benefits than most assume.

The link between sleep quality and life satisfaction highlighted in this paper might be bidirectional. A satisfied mindset about life may increase sleep quality, but as our findings imply, a good sleep may also affect how positively one evaluates his/her life. More conclusive statement about causal direction should be derived from additional longitudinal work. One notable finding is that the zero-sum belief mediates sleep quality and life satisfaction, even controlling for traits known to strongly influence happiness (e.g., neuroticism). It implies that believing happiness as a fixed, predetermined experience is psychologically deflating, above and beyond the predisposition to experience negative affect. Finally, replicating current finding with more sophisticated sleep measures (e.g., polysomnography) and with diverse samples will be desirable.

What constitutes a good life? Many people in modern society may shove a “good sleep” below other priorities, such as high status or income. However, our study suggests that this inconspicuous daily routine not only restores the body, but also elevates the mind’s view of life.

## Ethics Statement

This study was carried out in accordance with the recommendations of the Yonsei University Research Ethics Committee. The protocol was approved by the Yonsei University Institutional Review Board. Participants gave written informed consent in accordance with the Declaration of Helsinki.

## Author Contributions

JS and JK developed the study concept and design. JS collected the data and performed the data analysis under the supervision of JK and drafted the manuscript. All authors approved the final version of the manuscript for submission.

## Conflict of Interest Statement

The authors declare that the research was conducted in the absence of any commercial or financial relationships that could be construed as a potential conflict of interest.
